# Changes in the Crystallinity Degree of Starch Having Different Types of Crystal Structure after Mechanical Pretreatment

**DOI:** 10.3390/polym12030641

**Published:** 2020-03-12

**Authors:** Karina Dome, Ekaterina Podgorbunskikh, Aleksey Bychkov, Oleg Lomovsky

**Affiliations:** 1 Department of Natural Sciences, Novosibirsk State University, Pirogov Str. 1, Novosibirsk 630090, Russia; domekarina@ya.ru; 2 Institute of Solid State Chemistry and Mechanochemistry, Siberian Branch, Russian Academy of Sciences, Kutateladze Str. 18, Novosibirsk 630128, Russia; podgorbunskikh@solid.nsc.ru (E.P.); lomov@solid.nsc.ru (O.L.); 3 Department of business, Novosibirsk State Technical University, K. Marks Ave. 20, Novosibirsk 630073, Russia

**Keywords:** crystalline structure of starch, crystallinity degree, amorphization, mechanical activation, rate constant, energy expenditure

## Abstract

This paper examines the effect of mechanical activation on the amorphization of starch having different types of crystalline structure (*A*-type corn starch; *B*-type potato starch; and *C*-type tapioca starch). Structural properties of the starches were studied by X-ray diffraction analysis. Mechanical activation in a planetary ball mill reduces the degree of crystallinity in proportion to pretreatment duration. *C*-type tapioca starch was found to have the highest degree of crystallinity. Energy consumed to achieve complete amorphization of the starches having different types of crystalline structure was measured. The kinetic parameters of the process (the effective rate constants) were determined. The rate constant and the strongest decline in the crystallinity degree after mechanical activation change in the following series: *C*-type starch, *A*-type starch, and *B*-type starch.

## 1. Introduction

Starch is a high-molecular-weight plant-derived polysaccharide that consists of amylose and amylopectin and occurs in many crops, such as potato tubers, corn kernels, and cassava roots [1,2]. Starch is an affordable biologically renewable source of feedstock for the food, paper, pharmaceutical, and chemical industries [3,4,5]. Enzymes hydrolyze starch to its monomer glucose, which is an important and promising platform molecule employed in modern bioengineering [6]. Starch-based composites are used in food industry to develop various approaches for preserving and prolonging tastes and smells [7]. Chemical modification of native starch gives rise to products with tailored functional properties, which can be used for manufacturing of biodegradable packaging films, functional foods, etc. [8,9,10,11].

**Figure 1:**
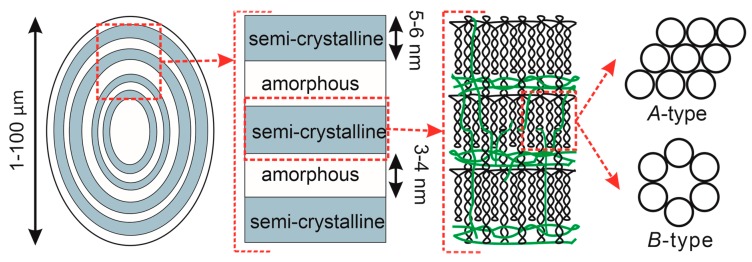
A graphical illustration of starch structure: from granules to glucosyl units; the double helices and branched segments of amylopectin are shown with black lines; single helices of amylose are shown with green lines; and circles denote the double helices in *A*- or *B*-polymorphic crystals (top view).

Starch consists of two types of polysaccharides. Amylose is a linear polymer, while amylopectin is a branched one. Figure 1 shows the starch structure, from granules to glucosyl units. The size of a schematic granule with growth rings extending from the hilum ranges from 1 to 100 μm [3]. The supramolecular complex of these polysaccharides forms granules that are partially crystalline [12,13]. A granule contains alternating semicrystalline and amorphous regions. The crystalline region consists of double helices of amylopectin, while the amorphous region is formed by amylose chains and branched segments of amylopectin. Amylose chains are interspersed among with amylopectin chains. The morphology, shape, size, degree of crystallinity and reactivity of starch depend on the origin and composition of the starch-bearing feedstock. 

The crystallinity depends on the degree of structural ordering in a solid body. The crystallinity of native starch granules typically is 14%–45%; that it why there are a number of challenges related to its use and processing [14]. Table 1 summarizes specific examples of starch isolated from different sources (typical crystallinity degrees are specified for them).

The materials produced from native starch are characterized by low heat stability, low water solubility, high gelatinization temperature, high viscosity, and poor mechanical properties. A starch with high crystallinity degree has low reactivity. Therefore, starch needs to be pretreated to ensure the energetic and economic feasibility of refining starch-containing biomass as this type of treatment improves the mechanical and rheological properties of starch.

Starch crystallinity is ensured by packing of amylopectin double helices in a unit cell. The double helix of amylopectin is formed by two polyglycoside chains. Several polymorphs characterized by different crystalline structures are differentiated: *A*-type, *B*-type, and *C*-type starches [18].

The *A*-type crystalline structure is typical of cereal starches (e.g., those derived from corn, wheat, and rice) [19]. In this case, the crystalline portion of starch consists of left-handed parallel-stranded double helices packed in a monoclinic unit cell (Figure 1), with the following lattice parameters: a = 11.90 Å, b = 17.70 Å, c = 10.52 Å, and α = β = γ = 90° [13]. The dense packing of the *A*-type crystal structure prevents chemical reactions (e.g., acid hydrolysis) from occurring. The *B*-type crystalline structure is typical of high-amylose starches contained in tubers, fruits, and stems (e.g., starches extracted from potatoes, bananas, canna, and sago) [19]. The crystalline portion of starch is formed by six left-handed parallel-stranded double helices packed in a relatively loosely packed hexagonal unit cell (Figure 1) with the following lattice parameters: a = b = 18.50 Å, c = 10.40 Å, α = β = 90°, and γ = 120° [13]. The *C*-type crystalline structure is found in starch extracted from legumes or plant roots (e.g., pea seeds or cassava roots) [19]. It is formed by coexisting *A*- and *B*-type crystallites: a *C*-type starch granule has a core with a *B*-type structure surrounded by *A*-type crystallites [18]. The lattice parameters for this starch type are as follows: a = b = 18.50 Å, c = 10.47 Å, α = β = 90°, and γ = 120° [13]. Hence, X-ray diffraction analysis is employed to identify the crystalline structure of starch under study.

Starch-containing biomass, which is often used in food industry, needs to be pretreated using the methods not involving chemical reagents. Mechanical pretreatment of natural plant-derived polymers is known to reduce grain size of a substance, thus increasing its reactivity in the subsequent heterogeneous processes [20,21,22]. Polymers undergo plastic deformation during mechanical pretreatment. Defect nucleation and accumulation causes disordering of the crystal structure. In particular, mechanical pretreatment of cellulose in a planetary ball mill gives rise to a reactive product that is characterized by disordered ultrastructure, increased specific surface area, and reduced crystallinity degree [23,24]. For starch, mechanical pretreatment also reduces the grain size and increases the content of the amorphous phase, making it possible to alter the hydrating and paste-forming properties of modified starch [25,26]. 

However, parameters strongly depending on the type of selected grinder and nature of the sample (e.g., pretreatment duration and rotor speed) are currently used to describe the processes of mechanical activation of the biomass. Grinding and mechanochemical processes are very energy-intensive, therefore, even minor optimization of these processes may have substantial economic effects [27,28,29,30]. Today, energy efficiency for mechanical disordering of starch is one of relevant questions [31].

The aim of this study was to investigate disordering of the crystalline structure of starch in three different polymorphic modifications caused by mechanical activation. The regularities “consumed energy—degree of amorphization” were also studied to determine which structural type of starch is least resistant to mechanical impact.

## 2. Materials and Methods 

Corn starch (state standard GOST 32159-2013; OOO “Garnec”, Russia), potato starch (state standard GOST R 53876-2010; OOO “Garnec”, Russia), and tapioca starch (state standard GOST 32159-2013; OOO “Garnec”, Russia), all of premium grade, were used as study objects. Moisture and ash contents in the samples were determined gravimetrically using the methods described in [32] and [33], respectively.

Particle size of the starches under study was determined by laser diffraction analysis on a Microsizer 201 particle size analyzer (VA Instalt, Saint Petersburg, Russia) equipped with an ultrasonic disperser.

The morphology of the selected starches was characterized by scanning electron microscopy. The data were recorded on a TM-1000 scanning electron microscope (Hitachi, Tokyo, Japan) at an accelerating voltage of 15 kV.

Mechanical activation of starch samples corresponding to different polymorphs was conducted in an AGO-2 laboratory-scale water-cooled planetary ball mill (grinding body acceleration, 200 m/s^2^; nominal motor power, 1.1 kW). Steel balls (diameter, 5 mm; weight, 200 g) were used as grinding media. The weight of treated material was 10 g; duration of mechanical activation was varied from 0 to 600 s.

Energy consumption for mechanical activation was measured using a Mercury high-speed wattmeter (Incotex Electronics Group, Moscow, Russia) interconnected with a DVP-SA2 industrial controller (Delta Electronics, Inc., Taipei, Taiwan) using the ModBus protocol.

The structural properties of starches were characterized by X-ray diffraction analysis on a D8 Advance powder diffractometer (Bruker, Karlsruhe, Germany) with monochromatic CuKα radiation in the Bregg–Brentano reflection geometry. Step size was 0.0195°. The analysis was conducted in a range of 2θ angles (3–70°) at a voltage of 40 kV and current of 40 mA. X-ray radiation wavelength was 1.5406 Å. No diffraction peaks were recorded at 2θ angles > 30°, which is consistent with the data published earlier [2,14,15,16,17]. In this connection, the range of 2θ angles of 3–30° was used for the sake of clarity.

**Figure 2:**
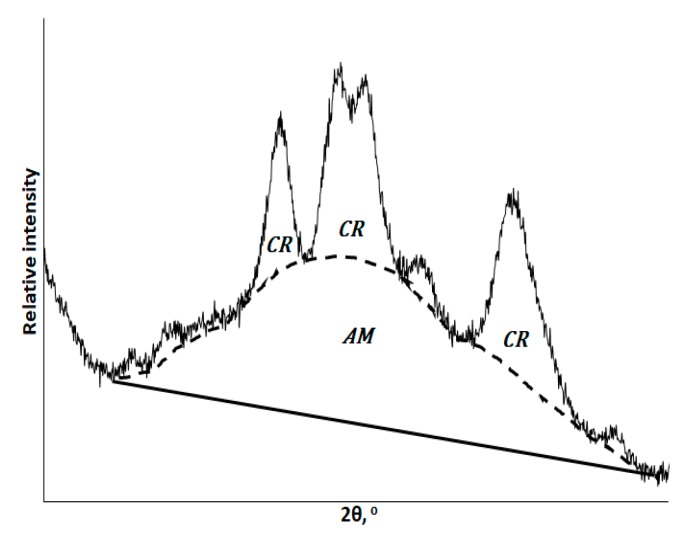
A graphical illustration of the method used to calculate the crystallinity degree of starch, where CR and AM denote the crystalline and amorphous phases, respectively.

The degree of crystallinity of starches under study was estimated using the method proposed by Nara and Komiya (1983) [12,15]. A smoothed curve connecting peak baselines was plotted on the XRD pattern. The areas above and under the smoothed curve corresponded to the crystalline and the amorphous portions of starch, respectively. The crystallinity degree was calculated as a ratio between the area corresponding to crystallites and the total area of the XRD pattern under the curve (Figure 2).

The crystallinity degree was calculated as the ratio between the area corresponding to the crystalline phase and the total area under the XRD curve using the formula:

(1) $$CI=\frac{S_{cr\cdot phase}}{S_{total}}\times 100\%,$$

where *CI* is the crystallinity degree; *S_cr.phase_* is the area corresponding to the crystalline phase; and *S_total_* is the total area under the XRD pattern.

The amorphization degree was calculated as the ratio between the change in the crystallinity degree and the initial crystallinity degree using the formula:

(2) $$AM=\frac{CI_{0}-CI_{t}}{CI_{0}},$$

where *AM* is amorphization degree; *CI_0_* is the initial crystallinity degree; and *CI_t_* is the crystallinity degree after mechanical pretreatment.

## 3. Results

Particle size (Table 2) was determined to characterize the selected starch samples. Particle size analysis demonstrated that potato starch has the largest granule size (43.1 µm). Corn and tapioca starches are characterized by close particle size (13.1 µm and 13.7 µm) and similar particle size distribution, which was unimodal for each starch type.

**Figure 3:**
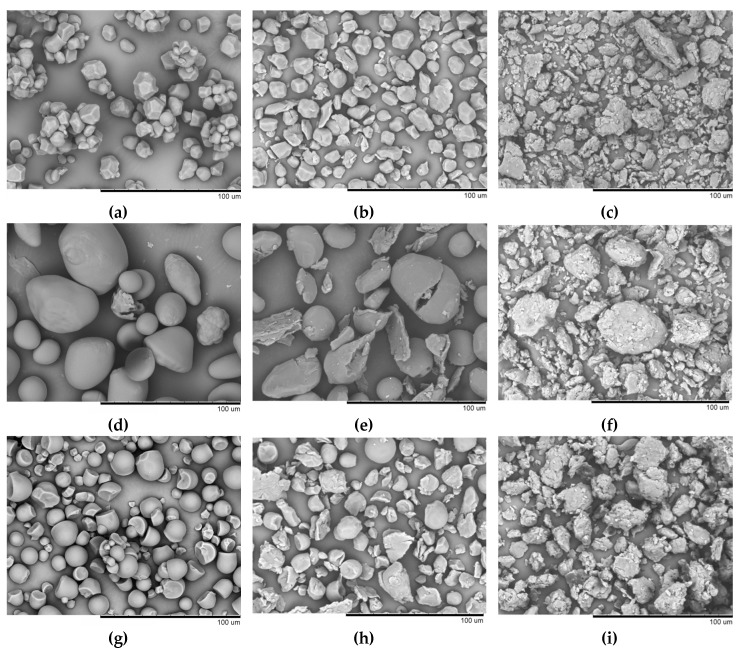
Micrographs of native granules and granules subjected to mechanical pretreatment for 60 and 600 s for corn (**a**–**c**), potato (**d**–**f**), and tapioca (**g**–**i**) starches.

Scanning electron microscopy (SEM) images of the investigated starches with different crystalline structures were recorded (Figure 3).

Corn starch granules (*A*-type) are irregular polygons with concave facets (Figure 3a). Potato starch granules (*B*-type) have a spherical, elliptical, or an irregular shape (Figure 3d). Grain surface is smooth (or is characterized by slight roughness, Figure 3g). The size of *C*-type tapioca granules is comparable to that of corn starch (diameter being <20 µm). The granules are predominantly spherical. The particles are irregularly shaped and have multiple facets due to natural mechanical defects. The surface of tapioca starch granules is smooth. Our findings on starch granule size are consistent with the earlier published laser diffraction spectrometry data [4]. After the mechanical pretreatment for 60 s, the grain shape for all three starch types changes due to the impact-shearing action. The mechanical pretreatment for 600 s resulted in complete degradation of corn and tapioca starch granules and particle aggregation. Some potato starch granules partially retained their initial spherical or elliptical shape. The degraded starch granules have a similar morphology regardless of their biological origin and type of the crystal structure. Hence, it was demonstrated by scanning electron microscopy that mechanical pretreatment of starch is accompanied by degradation of the structure of a starch granule. The revealed changes in granule morphology can be indirectly indicative of changes in the ultrastructure of the objects under study (including changes in the crystallinity degree).

**Figure 4:**
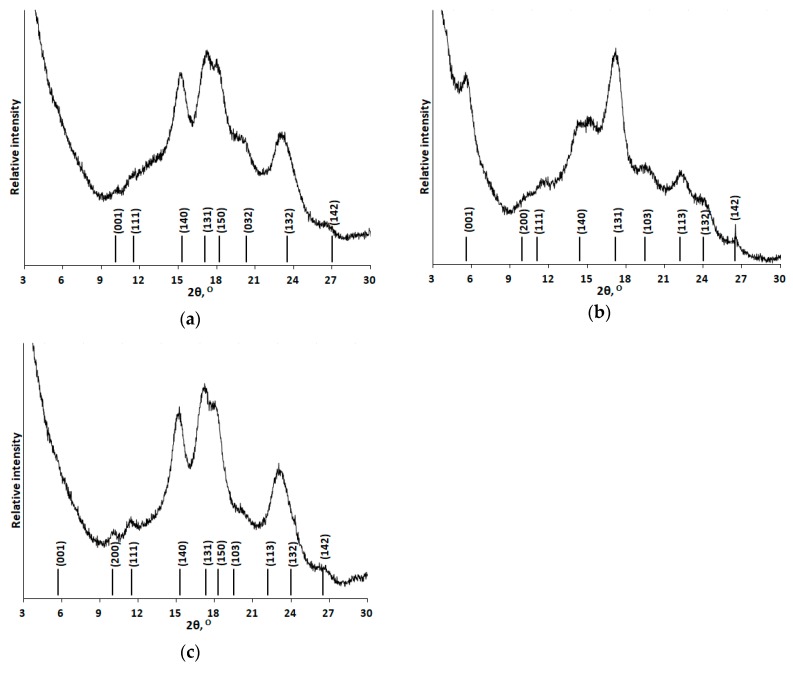
X-ray diffraction patterns of corn (**a**), potato (**b**), and tapioca (**c**) starches not subjected to mechanical activation. The positions of idealized reflections were taken from [34].

The X-ray diffraction patterns of corn, potato, and tapioca starches are shown in Figure 4. The XRD pattern of corn starch contains diffraction peaks (2θ) at 15.00°, 22.93°, and a doublet at 17.02° and 17.92°, which correspond to the *A*-type crystalline structure (Figure 4a) [14,34]. The XRD pattern of potato starch contains diffraction peaks (2θ) at 5.58°, 15.13°, 17.23°, 19.67°, 22.20°, 24.00°, and 26.35°, which correspond to the *B*-type crystalline structure (Figure 4b) [14,34]. The *C*-type crystalline structure consists of *A*- and *B*-type crystallites, so the XRD pattern can contain various superpositions of the characteristic diffraction peaks depending on the ratio between the contents of these polymorphs. Diffraction peaks (2θ) at 15.15°, 22.78°, and a doublet at 17.12° and 18.19 corresponding to the *C*-type crystalline structure are observed in the XRD pattern of tapioca starch (Figure 4c) [14,34].

**Figure 5:**
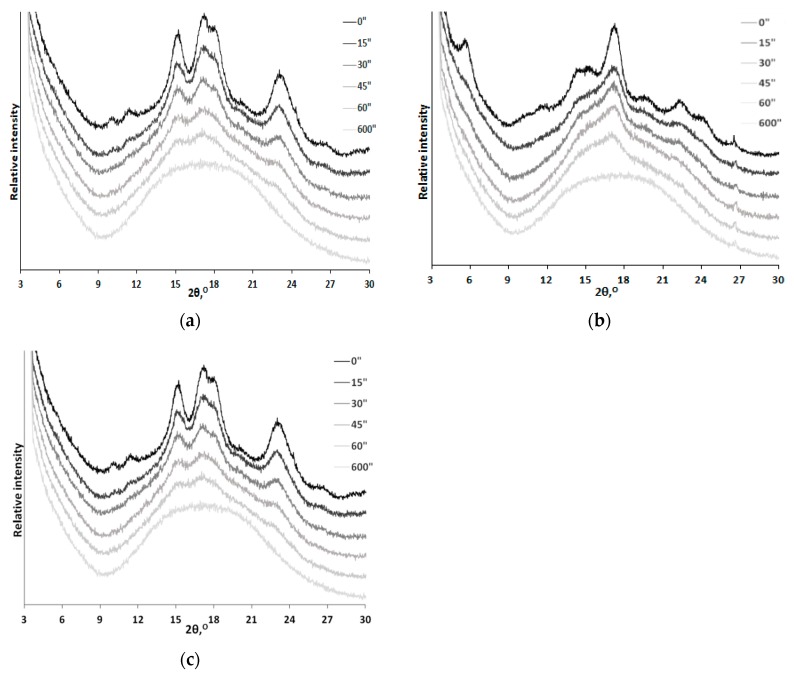
XRD patterns of corn (**a**), potato (**b**), and tapioca (**c**) starches after mechanical activation for 15–600 s.

Figure 5 shows the X-ray diffraction patterns of starches under study after they were subjected to mechanical activation in a planetary ball mill for 15–600 s. One can see that the intensity of reflections corresponding to crystallites contained in starch decreases with increasing duration of mechanical pretreatment. Mechanical activation causes disordering of the crystalline structure of starch. The amorphous phase no longer has long-range symmetry as can be seen from the absence of clear diffraction peaks in the XRD patterns. Therefore, X-ray diffraction patterns for completely amorphized samples subjected to pretreatment for 10 min were recorded. These samples were used as knowingly amorphous reference samples for calculating the crystallinity degree [23].

Table 3 lists the crystallinity degrees for the initial and mechanically activated starch samples. The XRD data indicate that the highest crystallinity degree at the initial time instant was observed for tapioca starch (42%), while the lowest one was recorded for potato starch (28%).

**Figure 6:**
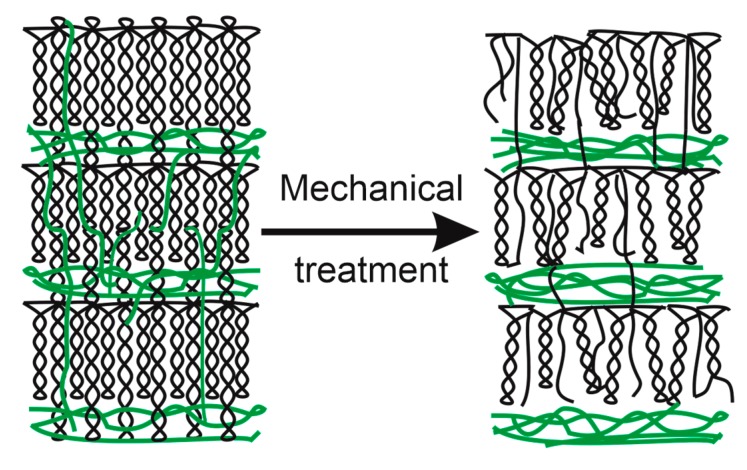
Schematic illustration of starch amorphization as a result of mechanical treatment.

The crystalline phase of starch predominantly consists of short-branched amylopectin chains. During mechanical pretreatment in a ball mill, some of mechanical energy acquired upon a collision is converted to the intrinsic energy of the sample. Some of excessive energy is consumed for defect formation. Mechanical pretreatment of starch samples led to defect accumulation in the crystal structure, thus causing the disruption of double helices of amylopectin and single helices of amylase due to the rupture of hydrogen or glycosidic bonds, as well as the release of interspersed amylose molecules into the amorphous phase (Figure 6) [10,26,35,36,37].

It was shown that longer duration of mechanical activation leads to gradual amorphization of the crystalline structure of *A*-, *B*-, and *C*-type starches, so their crystallinity degree decreases. In all of the cases, pretreatment for 60 s gave rise to the almost completely amorphized products (CI = 11%–14%). The greatest changes in the crystallinity degree were observed for tapioca starch (from 42% to 11%), while the smallest changes were revealed for potato starch (from 28% to 13%).

**Figure 7:**
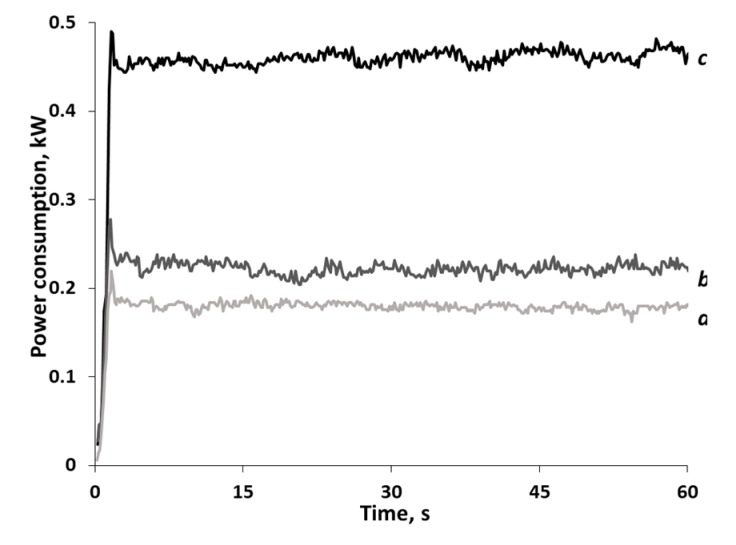
Actual power consumption of the planetary ball mill AGO-2: (**a**) the planetary gear without jars, (**b**) the planetary gear with empty jars; and (**c**) jars containing grinding bodies and starch.

A high-speed wattmeter was used to measure the power consumption of the planetary ball mill and energy expenditure for the experiment. Figure 7 shows the dynamics of changes in actual power consumption as the equipment is operated in various modes. One can see that power consumption during mechanical activation of the material is twice higher than that during operation of a mill with empty jars.

**Figure 8:**
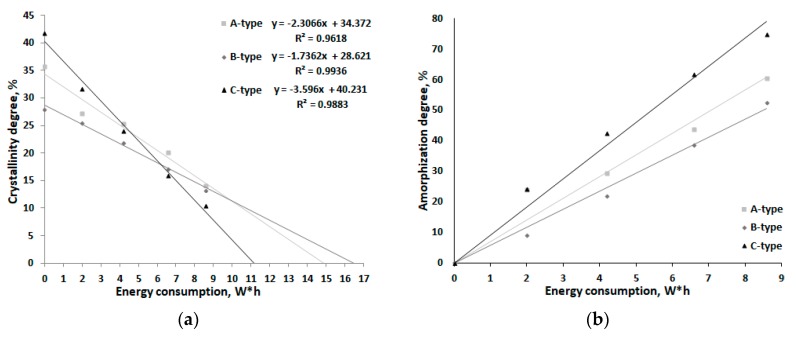
Changes in the crystallinity degree (**a**) and amorphization degree (**b**) of starches having different crystalline structures as functions of energy consumption during mechanical activation.

The diagrams illustrating changes in the crystallinity degree and normalized changes in the amorphization degree (which are visually more informative) are shown in Figure 8. One can determine that complete amorphization of corn, potato, and tapioca starches will require 16.5, 14.9, and 11.2 W*h, respectively.

To determine the kinetic parameters of starch amorphization, this process can be described using the kinetics of a pseudo-first-order reaction [37]:

(3) $$S_{cr}\to \;S_{am},$$

(4) $$\frac{d\left[S\right]}{dt}=-k\times \left[S\right],$$

(5) $$\ln\left(\;\frac{\left[S\right]}{{\left[S\right]}_{0}}\;\right)=-kt,$$

where [*S*] is the current content of the crystalline phase of starch; [*S*]_0_ is the initial content of the crystalline phase of starch; *t* is the duration of mechanical activation; and *k* is the effective rate constant of the process.

Table 4 lists the calculated effective rate constants of amorphization of starches having different crystalline structures. The *C*-type tapioca starch has the highest rate constant of amorphization, while those for *A*- and *B*-type starches are comparable and twice lower. The *C*-type crystalline structure is formed by coexisting *A*- and *B*-type crystallites. According to the concept regarding the nature of mechanical impact exerted on polymeric materials and composites, it is fair to assume that when mechanical impact is applied to an object with a heterogeneous structure (*C*-type starch), the resulting stress wave front propagates nonuniformly. Therefore, crystal structure disordering takes place at a faster rate than when processing an object having a homogeneous structure (*A*- and *B*-type starches). Mechanical pretreatment of homogeneous *A*- and *B*-type starches is less efficient because of the uniform propagation of the deformation wave. Therefore, it is fair to say that the *A*- and *B*-type starches are more resistant to mechanical impact. 

The crystallinity degree significantly affects the physicochemical properties such as hardness, density, transparency, and reactivity. However, these properties do not depend solely on crystallinity degree and are also affected by granule size and the type of crystal structure. Mechanical pretreatment results in particle refinement and increases the amount of the amorphous phase of starch, eventually improving the solubility and reactivity of the sample. These properties were employed when conducting enzymatic hydrolysis of mechanically pretreated biomass and developing novel types of functional foods characterized by improved nutrient bioavailability [38].

## 4. Conclusions

The effect of mechanical activation in a planetary ball mill on amorphization of starch depending on the type of its crystalline structure has been studied in this work. It has been shown that increasing duration of mechanical activation gives rise to samples with various degrees of crystallinity, as well as to the completely amorphized sample. Power consumption and energy consumed for mechanical activation have been measured. The energy consumed for amorphization of starch samples depends on the type of crystalline structure of starch. The lowest value was observed for potato starch (*B*-type structure), while the highest one, for tapioca starch (*C*-type structure). The effective rate constants, parameters depending on the crystalline structure of starch, were calculated in order to describe the amorphization kinetics. Tapioca starch having the *C*-type structure is characterized by the strongest reduction of its crystallinity degree after mechanical activation and the highest amorphization rate constant. Hence, mechanical activation of *A*-, *B*-, and *C*-type starches in a planetary ball mill under various conditions alters their degree of crystallinity, thus broadening the range of their industrial applications. The increased reactivity of starch was utilized to develop novel types of functional foods characterized by better nutrient bioavailability.

## Figures and Tables

**Table 1:** The crystallinity degree of starch obtained from different sources.

Type	Sources of Starch	Crystallinity Degree, %	References
*A*	corn	14–39	[2,15]
	finger millet	~30	[14]
	maize	~27	[14]
	wheat	27–36	[3,15]
	waxy rice	~38	[15]
*B*	potato	23–25	[14,15]
	banana	18–22	[16]
*C*	soybean	27–36	[17]
	green gram	~32	[14]
	cassava	~13	[2]
	tapioca	35–38	[14,15]

**Table 2:** Analysis of granule size for starches having different crystalline structure**.**

Sample	Moisture Content, %	Weighted Arithmetic Mean Diameter, µm	d_(0,1)_, µm ^1^	d_(0,5)_, µm ^1^	d_(0,9)_, µm ^1^
Corn Starch	7.2 ± 0.3	13.1 ± 0.1	3.5	12.5	18.9
Potato Starch	9.0 ± 0.5	43.1 ± 0.1	18.9	37.7	65.5
Tapioca Starch	8.1 ± 0.4	13.7 ± 0.1	3.6	12.5	18.9

^1^ d(0,1), d(0,5), d(0,9)—such a granule diameter value at which 10%, 50%, or 90% of all granules have a smaller diameter.

**Table 3:** The crystallinity degrees of starch depending on duration of mechanical activation.

Sample	Type	Crystallinity Degree, %					
		0 s	15 s	30 s	45 s	60 s	600 s
Corn Starch	*A*	36 ± 2	27 ± 2	25 ± 2	20 ± 1	14 ± 1	<10 ^2^
Potato Starch	*B*	29 ± 1	26 ± 1	22 ± 1	17 ± 1	13 ± 1	<10 ^2^
Tapioca Starch	*C*	42 ± 1	32 ± 1	24 ± 1	16 ± 1	11 ± 1	<10 ^2^

^2^ <10—completely amorphous.

**Table 4:** Effective rate constants of amorphization of starches having different crystalline structures.

Sample	Type	Effective Rate Constant of Amorphization, s^−1^
Corn Starch	*A*	15.5 × 10^−3^
Potato Starch	*B*	12.4 × 10^−3^
Tapioca Starch	*C*	23.1 × 10^−3^
